# Supplementary figures and images for: Validation of an algorithm to assess regular and irregular gait using inertial sensors in healthy and stroke individuals
Source: PeerJ. 2023 Dec 15;11:e16641. doi: 10.7717/peerj.16641 (PMC10726747; doi:10.7717/peerj.16641)

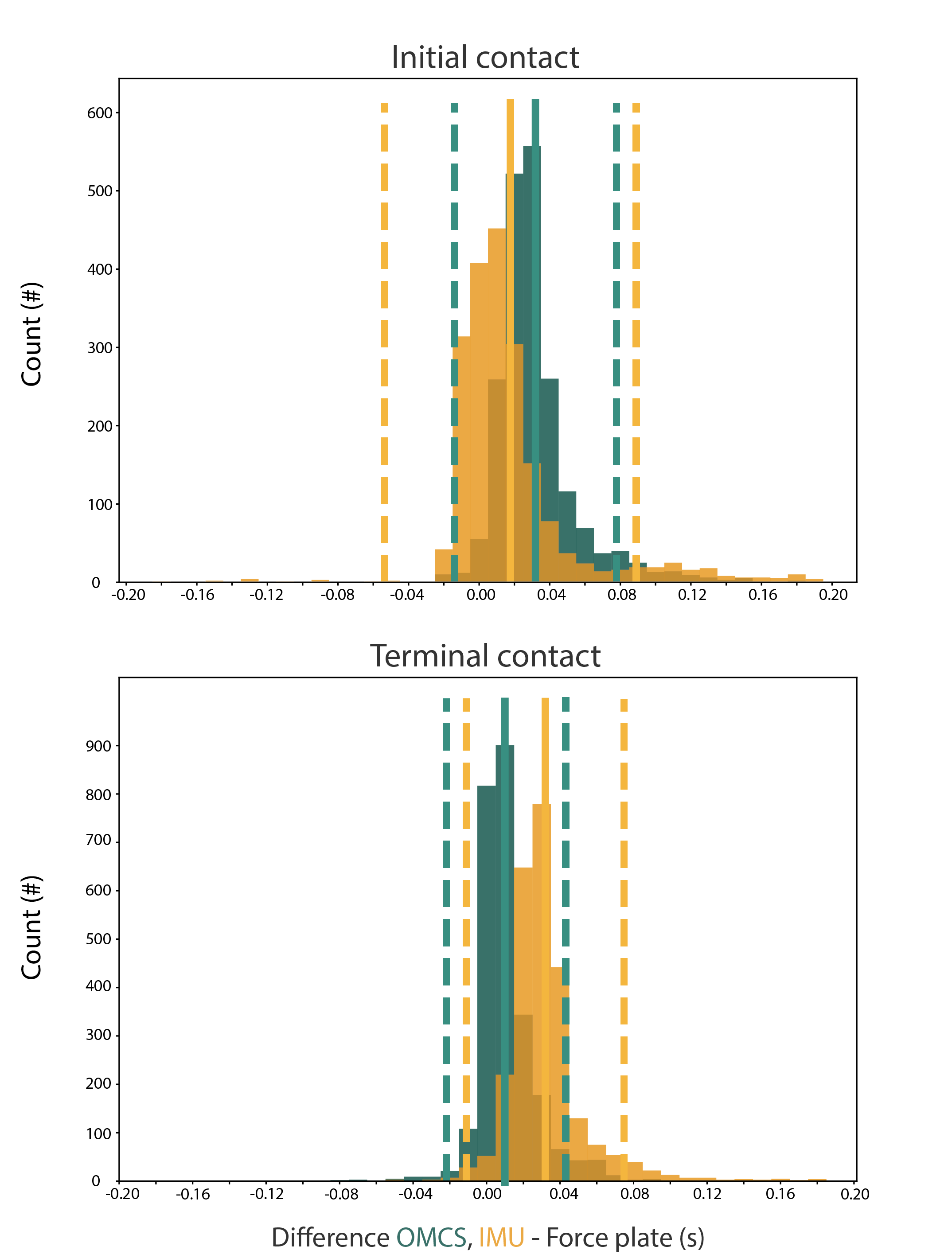

Supplement: Supplemental Information 1 — Histograms are on a stride-by-stride basis for all participants. Solid vertical lines indicate mean difference and dashed vertical lines indicate the 1.96*SD. [file peerj-11-16641-s001.png]
