# Supplementary material for: Validation of an algorithm to assess regular and irregular gait using inertial sensors in healthy and stroke individuals
Source: PeerJ. 2023 Dec 15;11:e16641. doi: 10.7717/peerj.16641 (PMC10726747; doi:10.7717/peerj.16641)
Supplement: Supplemental Information 2 — Mean differences were calculated as ‘IMU-based parameter –OMCS-based parameter’ and displayed under ‘mean’, while ‘SD’ notes the standard deviation of this difference. Trial ID’s starting with ‘STR’ are from stroke patients and ‘HC’ are from healthy controls. In regular walking, trail IDs containing ‘SP’ are at self-paced comfortable walking speed, while trial IDs containing ‘FS’ are at fixed-speed comfortable walking speed. In irregular walking, trial ID’s contain ‘SS’ indicating irregular walking induced by stepping stones. [file peerj-11-16641-s002.docx]

| **Regular walking** | | | | | | | **Irregular walking** | | | | | | |
| --- | --- | --- | --- | --- | --- | --- | --- | --- | --- | --- | --- | --- | --- |
| **Trial ID** | **Stride time (s)** | | **Stride length (m)** | | **Stride velocity (m/s)** | | **Trial ID** | **Stride time (s)** | | **Stride length (m)** | | **Stride velocity (m/s)** | |
|  | **mean** | **SD** | **mean** | **SD** | **mean** | **SD** |  | **mean** | **SD** | **mean** | **SD** | **mean** | **SD** |
| STR_01_SP | 0,00 | 0,01 | 0,01 | 0,03 | 0,01 | 0,02 | STR_01_SS | 0,00 | 0,02 | 0,01 | 0,02 | 0,01 | 0,01 |
| STR_02_SP | 0,00 | 0,08 | 0,02 | 0,14 | 0,01 | 0,06 | STR_02_SS | 0,00 | 0,03 | 0,01 | 0,07 | 0,00 | 0,06 |
| STR_03_FS | 0,00 | 0,07 | 0,01 | 0,04 | 0,01 | 0,01 |  |  |  |  |  |  |  |
| STR_04_SP | 0,00 | 0,04 | 0,02 | 0,06 | 0,01 | 0,02 | STR_04_SS | 0,00 | 0,04 | 0,02 | 0,05 | 0,01 | 0,02 |
| STR_05_SP | 0,00 | 0,01 | 0,00 | 0,02 | 0,00 | 0,02 | STR_05_SS | 0,00 | 0,01 | 0,00 | 0,02 | 0,00 | 0,01 |
| STR_06_SP | 0,00 | 0,01 | 0,00 | 0,02 | 0,00 | 0,02 | STR_06_SS | 0,00 | 0,01 | 0,00 | 0,02 | 0,00 | 0,01 |
| STR_07_SP | 0,00 | 0,01 | 0,01 | 0,02 | 0,01 | 0,02 | STR_07_SS | -0,06 | 0,01 | 0,01 | 0,02 | 0,01 | 0,01 |
| STR_08_SP | 0,00 | 0,03 | 0,00 | 0,02 | 0,00 | 0,02 | STR_08_SS | 0,01 | 0,10 | 0,00 | 0,04 | 0,00 | 0,03 |
| STR_09_FS | -0,01 | 0,08 | 0,00 | 0,06 | 0,01 | 0,04 | STR_09_SS | 0,00 | 0,07 | 0,00 | 0,06 | 0,01 | 0,05 |
| STR_10_SP | 0,00 | 0,01 | -0,03 | 0,02 | -0,03 | 0,02 | STR_10_SS | 0,09 | 0,01 | -0,02 | 0,02 | -0,02 | 0,01 |
| HC_01_SP | 0,07 | 0,01 | -0,05 | 0,03 | -0,05 | 0,03 | HC_01_SS | 0,05 | 0,01 | -0,08 | 0,05 | -0,08 | 0,04 |
| HC_03_SP | 0,00 | 0,01 | 0,00 | 0,02 | 0,00 | 0,02 | HC_03_SS | 0,00 | 0,01 | 0,01 | 0,02 | 0,01 | 0,02 |
| HC_04_SP | 0,09 | 0,01 | -0,05 | 0,02 | -0,05 | 0,02 | HC_04_SS | 0,05 | 0,01 | -0,04 | 0,02 | -0,04 | 0,02 |
| HC_05_SP | 0,08 | 0,01 | 0,00 | 0,02 | 0,00 | 0,02 | HC_05_SS | 0,00 | 0,01 | 0,00 | 0,02 | 0,00 | 0,02 |
| HC_06_SP | 0,04 | 0,01 | -0,02 | 0,02 | -0,02 | 0,02 | HC_06_SS | 0,00 | 0,01 | -0,01 | 0,03 | -0,01 | 0,03 |
| HC_07_SP | 0,05 | 0,01 | 0,00 | 0,01 | 0,00 | 0,01 | HC_07_SS | 0,05 | 0,01 | 0,00 | 0,02 | 0,00 | 0,01 |
| HC_08_SP | 0,00 | 0,01 | -0,05 | 0,03 | -0,05 | 0,03 | HC_08_SS | 0,00 | 0,01 | -0,05 | 0,04 | -0,05 | 0,04 |
| HC_09_SP | 0,00 | 0,01 | -0,14 | 0,04 | -0,12 | 0,04 | HC_09_SS | 0,00 | 0,01 | -0,14 | 0,06 | -0,13 | 0,05 |
| HC_10_SP | -0,05 | 0,01 | 0,00 | 0,02 | 0,00 | 0,01 | HC_10_SS | 0,00 | 0,01 | -0,01 | 0,02 | -0,01 | 0,02 |
| HC_11_SP | 0,05 | 0,01 | -0,08 | 0,03 | -0,07 | 0,02 | HC_11_SS | -0,05 | 0,01 | -0,09 | 0,05 | -0,08 | 0,04 |
| HC_12_SP | 0,05 | 0,01 | -0,01 | 0,01 | -0,01 | 0,01 | HC_12_SS | 0,00 | 0,01 | -0,01 | 0,03 | -0,01 | 0,02 |
| HC_13_SP | 0,10 | 0,01 | -0,03 | 0,02 | -0,03 | 0,01 | HC_13_SS | 0,00 | 0,01 | -0,03 | 0,03 | -0,02 | 0,02 |
| HC_14_SP | 0,00 | 0,01 | -0,01 | 0,02 | -0,01 | 0,02 | HC_14_SS | 0,00 | 0,01 | -0,02 | 0,03 | -0,02 | 0,03 |
| HC_15_SP | 0,05 | 0,01 | -0,06 | 0,05 | -0,06 | 0,04 | HC_15_SS | -0,05 | 0,01 | -0,05 | 0,06 | -0,05 | 0,05 |
| HC_16_SP | 0,08 | 0,01 | -0,03 | 0,02 | -0,04 | 0,02 | HC_16_SS | 0,09 | 0,01 | -0,02 | 0,03 | -0,02 | 0,02 |
| HC_18_SP | -0,04 | 0,01 | -0,03 | 0,02 | -0,03 | 0,02 | HC_18_SS | 0,04 | 0,01 | -0,02 | 0,02 | -0,02 | 0,02 |
| HC_19_SP | 0,00 | 0,01 | -0,03 | 0,02 | -0,03 | 0,02 | HC_19_SS | 0,05 | 0,01 | -0,02 | 0,01 | -0,02 | 0,01 |
| HC_20_SP | -0,04 | 0,01 | -0,03 | 0,03 | -0,03 | 0,03 | HC_20_SS | 0,05 | 0,01 | -0,03 | 0,03 | -0,03 | 0,03 |
| HC_21_SP | -0,05 | 0,01 | -0,01 | 0,01 | -0,01 | 0,01 | HC_21_SS | 0,00 | 0,03 | 0,00 | 0,03 | 0,00 | 0,03 |
| HC_22_SP | -0,09 | 0,01 | -0,06 | 0,04 | -0,06 | 0,04 | HC_22_SS | -0,05 | 0,01 | -0,02 | 0,04 | -0,02 | 0,03 |
