# Supplementary material for: Validation of an algorithm to assess regular and irregular gait using inertial sensors in healthy and stroke individuals
Source: PeerJ. 2023 Dec 15;11:e16641. doi: 10.7717/peerj.16641 (PMC10726747; doi:10.7717/peerj.16641)
Supplement: Supplemental Information 3 [file peerj-11-16641-s003.docx]

**Supplementary material 1**

**Post-hoc analysis: IMU-based and OMCS-based gait event detection against force plate-based gait event detection**

**Introduction**

Two methods are frequently used in literature to identify gait events; one being the OMCS-based method as used in this study, the other is based on force plate data [1–4]. The benefit of OMCS-based gait event detection, is that multiple strides per stretch in the overground lab can be analyzed, against only one stride per stretch on force plates.

To maximize the amount of strides for analysis in the overground lab, and be consistent in the used methods, the IMU-based algorithm was validated against OMCS in both settings. Nevertheless, during all treadmill walking trials force data was collected by the embedded force plates of the GRAIL. To check the magnitude of the difference including limits of agreement (LoA) at 1.96 standard deviation in gait event detection between the OMCS-based method used in this study and force plate data as ground truth, post-hoc analysis was performed.

**Methods**

All code for this post-hoc analysis is included in the scripts available from: <https://github.com/SintMaartenskliniek/IMU_GaitAnalysis> (Release ‘Validation study, tag v1.1.0’). Force plate data from all GRAIL trails was filtered by a forth order, zero shift Butterworth filter with cut-off frequency 20 Hz and down sampled to 100 Hz. Gait event detection was done based on a 10 Newton threshold. IC events were identified at the first instance the vertical force exceeded the threshold for at least 0.4 seconds, while TC events were identified at the first instance the vertical force was less than the threshold for at least 0.4 seconds.

Not all gait events could be identified based on the force plate data (participants did not always place their right foot on the right force plate and their left foot on the left force plate). Therefore, it was assumed that if a gait event was detected by both force plates and OMCS or IMU-based algorithms, they would be within a 0.2 second time window. Gait events within this window were then compared on instance of detection by histograms of the difference.

**Results**

OMCS detected IC 0.03 s [LoA: -0.01; 0.07] and TC 0.01 s [LoA: -0.03; 0.05] after the force plates. IMU-based analysis detected IC 0.02 s [LoA: -0.06; 0.10] and TC 0.03 s [LoA: -0.01; 0.07] after the force plates. Histograms of the difference between these methods are shown in Supplementary Figure 1.

<<INSERT SUPPLEMENTARY FIGURE 1 HERE>>

**Supplementary material 2**

**Mean difference between IMU-based and OMCS-based analyses for each subject, for each trial**

<<INSERT SUPPLEMENTARY TABLE 1 HERE>>
